# From Misinformation to Public Distrust: Urgency of Evidence‐Based Transparency, Communication and Community Engagement for Bangladesh's Typhoid Vaccination

**DOI:** 10.1002/hsr2.72860

**Published:** 2026-07-20

**Authors:** Md. Salauddin, Antu Biswas

**Affiliations:** ^1^ Department of Microbiology and Public Health Khulna Agricultural University Khulna Bangladesh

**Keywords:** Bangladesh, community engagement, health communication, health policy, trust, typhoid fever, vaccination hesitancy

## Abstract

**Background and Aims:**

In Bangladesh, typhoid fever poses a significant threat to public health and has been a major epidemic for many years, especially affecting children from low‐ and middle‐income families due to poor water, sanitation, and seasonal living conditions. This article highlights the nationwide Typhoid Conjugate Vaccine (TCV) campaign launched in October 2025 as a highly effective, cost‐efficient intervention to provide preventative measures and control antibiotic resistance.

**Methods:**

We searched PubMed, Scopus, Web of Science, and Google Scholar, alongside gray literature from WHO, Gavi, FAO, UNICEF, DGHS, and other Government of Bangladesh sources. Relevant evidence was synthesized to examine misinformation, public trust, and communication challenges surrounding typhoid vaccination in Bangladesh.

**Results:**

This perspective identifies major challenges to optimal vaccine coverage, specifically misinformation, vaccine hesitancy, and gaps in risk communication that must be addressed. To ensure long‐term vaccine acceptance and permanent typhoid control, the study emphasizes the necessity of educating the public through evidence‐based communication, community participation, and trust‐centered communication techniques.

**Conclusion:**

It is important for these communication strategies to be implemented to realize optimal coverage and ensure the control of typhoid is permanent in Bangladesh.

## 1 Introduction

Typhoid Fever (*Salmonella enterica serovar* Typhi) mostly affects the children under 15 years old disproportionately worldwide. It primarily affects low‐ and middle‐income countries like South Asia, Southeast Asia, and Sub‐Saharan Africa [1]. This fever causes 21.7 million cases and 200,000 deaths globally each year, with Bangladesh experiencing one of the world's highest incidence rates at 290 cases per 100,000 population. Bangladesh faces approximately 477,518 typhoid cases and 8,000 deaths annually (Burden of Typhoid Fact Sheet, 2024), with children under 15 accounting for 68% of fatalities. The October 2025 nationwide TCV campaign aims to vaccinate 50 million children (aged 9 months to 15 years) to curb this preventable disease [TCV Fact Sheet, 2025; Gavi [2, 3]].

## 2 Epidemiological Context

In Bangladesh, typhoid incidence has shown no consistent decline over the past 15 years, remaining around 100–300 cases per 100,000 population across studies. Variations are largely driven by seasonality and antimicrobial resistance patterns rather than sustained reduction, despite surveillance and vaccination efforts [4]. The highest reported incidence was 1,135 per 100,000, predominantly among children aged 5–9 years [5]. This higher figure reflects a localized outbreak peak rather than a national annual average, while a lower estimate of 290 per 100,000 was reported in 2021, with cases distributed across all age groups (< 5, 5–14, 15–49, and > 50 years). Between 2001 and 2014, 7100 culture‐confirmed *S*. Typhi cases were documented. During the pandemic period, incidence again peaked at 1,135 per 100,000 in 2021 [6, 7].

## 3 TCV Campaign Overview

With the epidemiological burden established, we now turn to the TCV campaign itself. On 12 October 2025, Bangladesh launched a nationwide TCV campaign with support from UNICEF, Gavi, and WHO, targeting ~50 million children aged 9 months to < 15 years with a single dose (Gavi). Despite WHO prequalification, Phase III trial evidence showing ~85% efficacy in > 41,000 Dhaka children (Gavi, 2021), and successful implementation in Pakistan, Nepal, Zimbabwe, and India, challenges persist. Pre‐launch registration reached only 33% (~18 million), with lower coverage in urban slums, alongside COVID‐19–era mistrust, misinformation, and limited local long‐term data (> 5 years) [7].

## 4 Challenges: Misinformation and Hesitancy

TCVs offer dual benefits: direct protection against disease and indirect control of antimicrobial resistance. By preventing infections, TCVs reduce unnecessary antibiotic use—currently, 25 suspected cases are treated with antibiotics for every true typhoid case. With Bangladesh confronting extensively drug‐resistant (XDR) strains [7] and WHO listing typhoid as a high‐priority pathogen, vaccination represents our most powerful tool against both disease and resistance [8, 9].

Despite strong epidemiological evidence and WHO prequalification, the campaign faces significant resistance: initial pre‐registration reached only 33% in some areas, driven by COVID‐19‐era distrust, with 46% citing side‐effect fears and social media misinformation. False claims circulate that TCVs are ‘experimental,’ lack safety data, contain live typhoid bacteria, or target poor children as ‘guinea pigs’ and represent ‘human experimentation’ simply because they are provided free—narratives that ignore robust clinical evidence (85% efficacy demonstrated in over 41,000 Dhaka children) from Bangladesh itself (Counterpoint, 2025). Also, targeting 491,736 children living in camps across Ukhiya and Teknaf. 410,345 children had received TCV, achieving 86.3% coverage (WHO, 2025). But the actual scenario is that the total number of children (below 18 years old) is approximately 59 million in 2023 in Bangladesh, but they claim their vaccination coverage was 97%, which was 42 million (UNICEF, 2025). The fact that the total population is roughly 175.7 million as of 2025 and the fertility rate is around 2. So, a huge population is outside of this coverage. To clarify: the 97% coverage (42 million) refers only to the campaign's target group, while the 59 million represents the total child population under 18 years in Bangladesh. The difference highlights genuine gaps in campaign reach. Typhoid is endemic, but adults are at risk too. This vaccination campaign is good, but it should be continued because Bangladesh is not only a densely populated country but also its hygiene practices and water management facilities are very poor—where approximately 41.7% of water sources were contaminated with fecal indicator bacteria (*E. coli*), increasing to around 61.7% at the point of consumption [10]. Currently, an extensively drug‐resistant typhoid strain exists in Bangladesh, which is alarming. As a result, treatment cost is increasing from around 5,450 BDT in 2020 to approximately 8,000 BDT in 2025, and also unwanted deaths may appear [UNICEF, 2025 [7]].

The challenges described above are not unique to TCV. A historical pattern exists. Despite the availability of the free COVID‐19 vaccine, the Human Papilloma Virus (HPV) vaccine, and other vaccination programs in Bangladesh, public uptake has often been suboptimal. Misinformation, myths, and rumors—combined with gaps in risk communication and public awareness—have significantly undermined vaccine acceptance. A similar pattern is now evident in the TCV campaign, where community response remains lower than expected despite the public health importance. Why does misinformation persist despite previous campaigns like those for COVID‐19 and HPV? Because past efforts focused on service delivery, not trust‐building. Communities received vaccines but not transparent explanations about side effects, manufacturing, or long‐term data. That gap enabled rumors to proliferate in the absence of authoritative information. The pattern repeats because the root cause‐institutional distrust‐remains unaddressed.

## 5 Critical Observation

The root causes of vaccine hesitancy in Bangladesh are layered. Socio‐culturally, rumors spread faster than facts in close‐knit communities where word‐of‐mouth carries more weight than official announcements. Politically, past vaccine campaigns have sometimes lacked transparency about procurement and adverse event reporting, which feeds suspicion. Digitally, social media platforms like Facebook amplify false claims—such as TCVs being ‘human experimentation’—without fact‐checking mechanisms. Similar patterns have been documented in Pakistan and India, where distrust led to polio vaccine boycotts. In Pakistan, attacks on vaccination workers followed false rumors of sterility [11]. In India, the HPV vaccine campaign collapsed due to inadequate community engagement [12]. Bangladesh can learn from these failures: trust cannot be presumed; it must be systematically cultivated through transparency and local dialog.

## 6 Recommendations and Policy Implications

This highlights an urgent need to develop and adopt evidence‐based vaccination policies that go beyond service delivery and actively build public trust. Strengthening transparency, improving risk communication, and addressing community concerns must be central components of future vaccination strategies to ensure sustainable success. The Government of Bangladesh, through the Directorate General of Health Services (DGHS), should initiate national policy dialogs involving key policymakers, public health experts, and stakeholders to address these challenges holistically. Incorporating vaccine education into school and madrasah curricula can foster early understanding of vaccine benefits and safety. Finally, persistent gaps in the TCV campaign should be addressed through targeted mass media communication, structured educational programs, engagement with grassroots populations, and strong community involvement. While DGHS operates these initiatives under the Expanded Programme on Immunization (EPI), current efforts remain insufficient and require strategic strengthening to effectively overcome vaccination challenges in Bangladesh. A more integrated, trust‐centered, and community‐responsive approach ‐ incorporating dedicated budgets and healthcare training ‐ is therefore urgently needed.

## 7 Conclusion

When public trust fails due to rumors or lack of manufacturing transparency, vaccination refusal follows. That refusal does not end with the individual. It opens the door to the next outbreak, which brings two predictable outcomes: unnecessary medical costs and avoidable deaths. Once that happens, the situation becomes exceedingly difficult to control. Therefore, we recommend active engagement from three groups: health policy makers, community members (through genuine public participation), and public–private partnerships, supported by responsible media partners. To make trust‐building operational, we propose two concrete steps: first, train local women as Community Health Workers to provide door‐to‐door vaccine education; second, add vaccine science to the school curriculum so that students learn how vaccines work. In the absence of such measures, no vaccination campaign is likely to achieve sustainable success.

## Author Contributions

**Md. Salauddin:** conceptualization, investigation, writing – original draft, writing – review and editing, supervision, validation, visualization, resources, data curation, methodology, software, project administration. **Antu Biswas:** writing – original draft. Both of the authors read and approved the manuscript.

## Funding

The authors have nothing to report.

## Conflicts of Interest

The authors declare no conflicts of interest.

## Transparency Statement

The lead and corresponding author, Md. Salauddin affirms that this manuscript is an honest, accurate, and transparent account of the study being reported; that no important aspects of the study have been omitted; and that any discrepancies from the study as planned (and, if relevant, registered) have been explained.

## Data Availability

Data sharing not applicable to this article as no datasets were generated or analyzed during the current study.
